# Assessment of vascularized free fibula transplantation revealing a congenital aplastic posterior tibial artery: a case report

**DOI:** 10.1186/1752-1947-8-75

**Published:** 2014-02-26

**Authors:** Takako Kanatani, Issei Nagura, Ikuo Fujita, Takuya Fujimoto, Masatoshi Sumi

**Affiliations:** 1 Department of Orthopaedic Surgery, Kobe Rosai Hospital, 4-1-23, Kagoike-dori, Chuo-ku, Kobe 651-0053, Japan; 2 The Department of Orthopaedics of Hyogo Cancer Center, 13-70, Kitaoji-Cho, Akashi 673-8858, Japan

**Keywords:** Magnetic resonance angiography, Peroneal artery, Vascularized free fibula transplantation

## Abstract

**Introduction:**

Anatomical abnormalities in the lower limb vessels are uncommon. However, the preoperative evaluation of the anatomical variations is very important for planning the operation procedure to prevent jeopardizing the donor leg.

**Case presentation:**

In this case report, a 23-year-old Asian woman who was scheduled to have vascularized free fibula transplantation for reconstruction of her wrist after excision of bone tumor in her distal radius, was found to have congenital aplastic posterior tibial arteries in both legs. These findings were found on magnetic resonance angiography (our preferred methodology due to its simplicity). We planned testing the sufficiency of her pedal pulses after temporarily clamping her peroneal artery but prior to harvesting, to ensure minimal risk to the longevity of her donor leg. During the operation, after dissection of a 10cm segment of her fibula with the peroneal artery, the peroneal artery proximal to the graft was temporarily clamped and the tourniquet was released. As adequate sustainable pedal pulses were confirmed, the graft was harvested and transplanted to her wrist. There was no morbidity in her right leg postoperatively and the union of the grafted fibula was substantiated 10 months postoperatively.

**Conclusions:**

We concluded two findings: firstly, for accurate preoperative planning of a vascularized free fibula procedure, examination of the bilateral lower leg vasculature either by angiography or other imaging should be performed. Secondly, abnormalities are not in themselves reason to abandon the vascularized free fibula procedure. We contend that pedal pulses should be evaluated preoperatively and provided that adequate foot circulation can be confirmed (by temporarily clamping the vessels and releasing the tourniquet during the operation prior to harvesting the free vascularized fibula) the procedure should be successful without jeopardizing the donor leg.

## Introduction

A vascularized free fibula transplantation is useful to reconstruct large segmental bone defects (>6cm) of the upper extremity [1-3]. An anatomical assessment of bilateral lower leg vasculature either by angiography or magnetic resonance angiography (MRA) is recommended prior to a vascularized free fibula procedure [4-7]. Although anatomical abnormalities in the lower limb vessels are uncommon, angiographic abnormalities have been reported at the rate of 3% to 25% [4-6,8] in different situations. These showed variations of branching of the popliteal artery with only two or even one main artery supplying the foot with either the posterior tibial, the anterior tibial or both being hypoplastic-aplastic.

This report describes a successful vascularized free fibula transplantation to the wrist (reconstruction after the excision of bone tumor) in which the donor leg was not jeopardized, where a congenital aplastic posterior tibial artery (PTA) in the donor lower leg was detected.

## Case presentation

**Figure 1:**
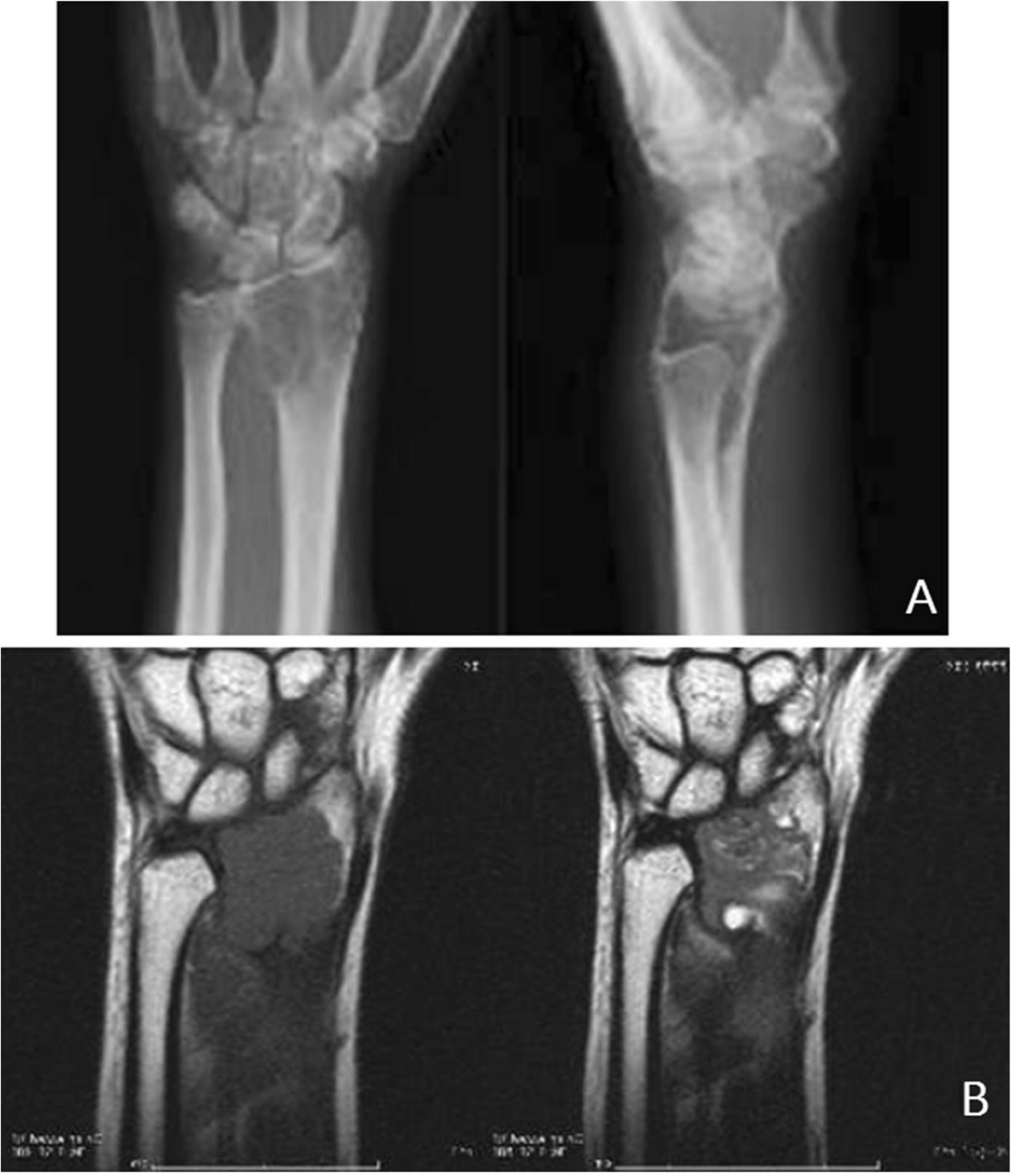
The preoperative X-ray (A) and magnetic resonance imaging (B) at the first visit.

**Figure 2:**
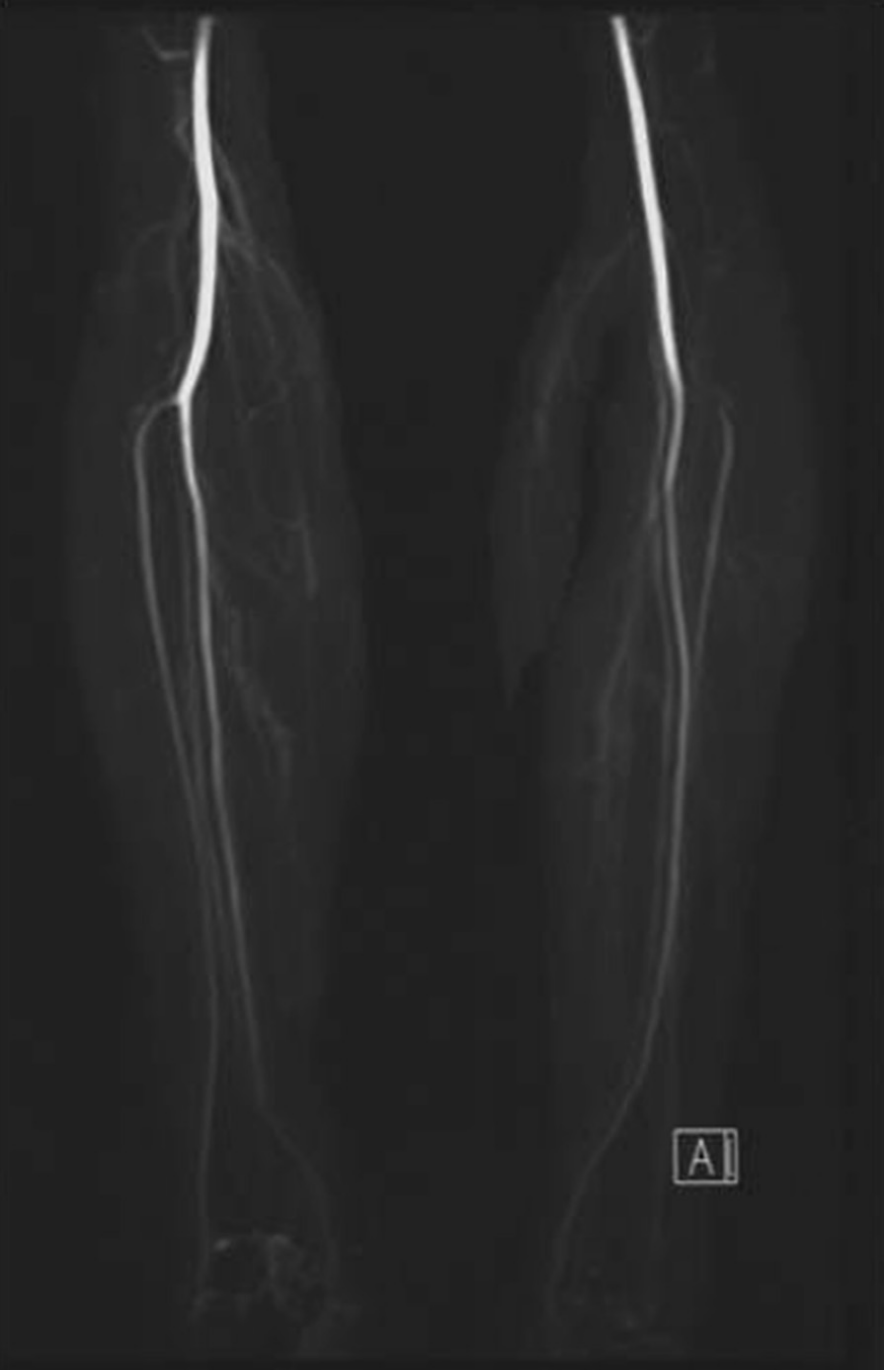
**The magnetic resonance angiography findings of the aplastic posterior tibial arteries in both lower legs.** The peroneal arteries replaced the posterior tibial arteries at the medial malleolus; right leg (left) and left leg (right).

A MRA of a 23-year-old Asian woman who was scheduled for a vascularized free fibula transplantation to reconstruct her left wrist after excision of a giant cell tumor in her distal radius (Figure 1A and B) showed a congenital aplastic PTA in her right lower leg (Figure 2). Her peroneal artery (PA) replaced the PTA at the medial malleolus and a similar finding was displayed in her left leg (Figure 2). In addition, her pedal pulses on her left foot were not palpable (due to previous knee trauma) making this fibula inappropriate as a donor section. In preoperative planning, a 10cm segment of bone graft was required for partial wrist fusion. It is generally recognized that a foot survives with anterior tibial artery only without PTA if the pedal pulses are maintained. Therefore we planned to confirm the pedal pulses before harvesting the graft to prevent jeopardizing her lower leg. We advised her of the risks involved and explained the precaution of testing the sufficiency of her pedal pulses after temporarily clamping her PA prior to harvesting, to ensure minimal risk to the longevity of her donor leg. If it was found that there was insufficient palpability of her pedal pulses then the procedure would be aborted.

**Figure 3:**
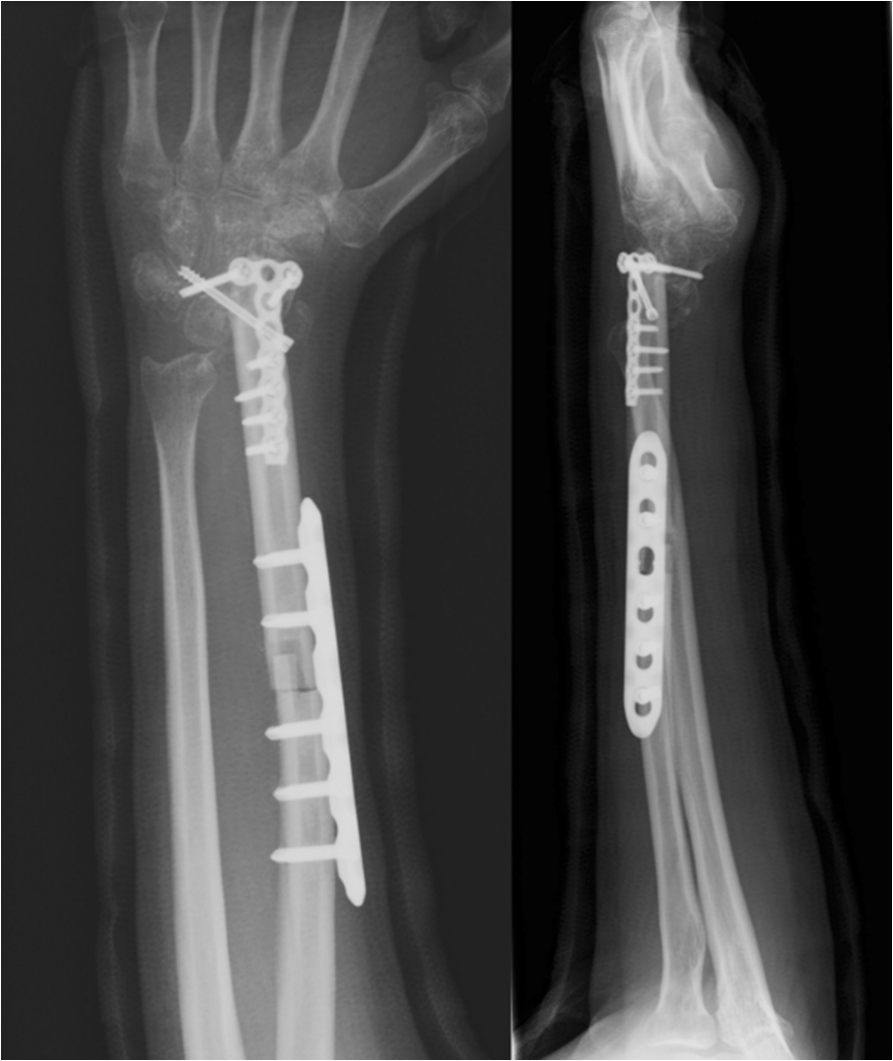
A 10cm segment of fibula bone graft was used for partial wrist fusion.

During the operation, after dissection of a 10cm segment of her fibula with the PA, the PA proximal to the graft was temporarily clamped and the tourniquet was released. As adequate sustainable pedal pulses were confirmed, the graft was harvested and transplanted to her wrist (Figure 3). There was no morbidity in her right leg postoperatively and the union of the grafted fibula was substantiated 10 months postoperatively.

## Discussion

Anatomical abnormalities in the lower limb vessels are uncommon. Strauch and Yu reported the anatomical variations of the PA and classified four different types [9]. According to their classification, our case is classified as Type D; the PA took the place of the PTA in 8% of all cases. However, Pototschnig *et al*. analyzed the intraoperative variations of the PA of 104 cases and classified four variations [10] in which there was no case that presented as “Type D” by Strauch *et al*. Angiographic abnormalities have been variously reported at the rate of 5.6% [4], 15.8% [5], 25% [6] and 3% [8] in different situations. These show variations of branching of the popliteal artery with only two or even one main artery supplying the foot with either the tibial posterior, the tibial anterior or both being hypoplastic-aplastic. Most reports recommend a routine preoperative anatomical assessment of bilateral lower leg vasculature either by angiography, by MRA or by ultrasonic Doppler flow meter before a vascularized free fibula or fibula flaps procedure [4-7,9]. Young *et al*. altered the operation plan in all 25% of cases planned for fibula harvest where angiographic abnormalities were detected [6]. However, we consider preoperative angiographic abnormalities in themselves an insufficient reason to abandon the vascularized free fibula procedure. We note that Lutz *et al*. reported the possibility of a false positive angiography due to vascular spasms and they concluded that clinical evaluation of pedal pulses was superior to angiography as a diagnostic tool in these cases [8].

Regarding the choice of the operation procedure, wrist arthroplasty with an articular fibular head graft was an option. However, the long-term clinical and radiographic results of the partial wrist arthrodesis with fibula bone for giant cell tumors of the distal radius were superior to arthroplasty with an articular fibular head graft [11], where wrist arthroplasty presented palmar subluxation of the carpal bones and degenerative changes in all cases. Also, Chung *et al*. reported that half of their cases presented those changes during the long-term follow-up period [12]. We selected the partial wrist arthrodesis because of the patient’s age of 23 years.

## Conclusions

Consequently we conclude two findings: firstly, that for accurate preoperative planning of a vascularized free fibula procedure, examination of the bilateral lower leg vasculature either by angiography or other imaging should be performed. We suggest MRA would be the preferred methodology due to its simplicity in providing the required data. Secondly, where abnormalities are detected, pedal pulses should be evaluated preoperatively. Then provided adequate foot circulation can be confirmed by temporarily clamping the vessels and releasing the tourniquet during the operation prior to harvesting the free vascularized fibula, the procedure should be successful.

## Consent

Written informed consent was obtained from the patient for publication of this case report and accompanying images. A copy of the written consent is available for review by the Editor-in-Chief of this journal.

## Abbreviations

MRA: Magnetic resonance angiography; PA: Peroneal artery; PTA: Posterior tibial artery.

## Competing interests

We declare that we have no competing interests.

## Authors’ contributions

TK and IN were major contributors in harvesting the vascularized free fibula and in writing the manuscript. IF and TF analyzed and interpreted the patient data and performed the excision of the tumor and bone fixation of the fibula. MS performed the analysis of MRA and magnetic resonance imaging. All authors read and approved the final manuscript.
